# Genetically Engineered‐Cell‐Membrane Nanovesicles for Cancer Immunotherapy

**DOI:** 10.1002/advs.202302131

**Published:** 2023-07-06

**Authors:** Qinzhen Cheng, Yong Kang, Bin Yao, Jinrui Dong, Yalan Zhu, Yiling He, Xiaoyuan Ji

**Affiliations:** ^1^ Jinhua Municipal Central Hospital Jinhua 321000 China; ^2^ Academy of Medical Engineering and Translational Medicine Medical College Tianjin University Tianjin 300072 China; ^3^ Medical College Linyi University Linyi 276000 China

**Keywords:** cancer immunotherapy, cell membrane nanovesicles (CMNs), genetically engineered‐cell‐membrane nanovesicles (GCMNs), genetic engineering, immune targets

## Abstract

The advent of immunotherapy has marked a new era in cancer treatment, offering significant clinical benefits. Cell membrane as drug delivery materials has played a crucial role in enhancing cancer therapy because of their inherent biocompatibility and negligible immunogenicity. Different cell membranes are prepared into cell membrane nanovesicles (CMNs), but CMNs have limitations such as inefficient targeting ability, low efficacy, and unpredictable side effects. Genetic engineering has deepened the critical role of CMNs in cancer immunotherapy, enabling genetically engineered‐CMN (GCMN)‐based therapeutics. To date, CMNs that are surface modified by various functional proteins have been developed through genetic engineering. Herein, a brief overview of surface engineering strategies for CMNs and the features of various membrane sources is discussed, followed by a description of GCMN preparation methods. The application of GCMNs in cancer immunotherapy directed at different immune targets is addressed as are the challenges and prospects of GCMNs in clinical translation.

## Graphical abstract

Enabled by recent advances in nanotechnology and materials science, genetically engineered‐cell‐membrane nanovesicles (GCMNs) hold great potential to provide better solutions for cancer immunotherapy. By reviewing the state‐of‐the‐art GCMNs and highlighting the promising technology development, this review intends to spark further research and development activity in this critical research area.

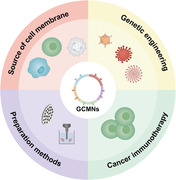

## 1 Introduction

Cancer is a major threat to human life and health. Current clinical treatments for cancer, such as surgery, chemotherapy, and radiotherapy, have inherent limitations.^[^ ^1^ ^]^ However, with the rapid development of molecular biology and cancer genomics, a deeper understanding of the signaling pathways related to the human immune system has been achieved, resulting in the remarkable therapeutic effects of cancer immunotherapy in clinical practice.^[^ ^2^ ^]^ Cancer immunotherapy activates T cells, dendritic cells (DCs), macrophages, natural killer cells, and other immune cells to generate an anticancer immune response, thus controlling and eradicating primary and distant cancers.^[^ ^3^ ^]^ In recent years, several cancer immunotherapies have emerged, including immune‐checkpoint inhibitors (ICIs), cytokine therapy, chimeric antigen receptor (CAR)‐T cell therapy, and cancer vaccines. Among these, ICIs that target the programmed cell death protein 1 (PD‐1)–PD‐1 ligand (PD‐L1) immune checkpoint have achieved significant clinical results.^[^ ^4^ ^]^ Many studies have demonstrated that strategies aimed at targeting immune targets on different cells can greatly enhance efficacy and reduce toxic side effects.^[^ ^5^ ^]^

Cell‐membrane‐derived nanovesicles referred to nanoscale phospholipid bilayer vesicles, which are prepared by direct secretion or physicochemical methods. Due to their excellent biocompatibility, cell‐membrane‐derived nanovesicles have become a favored drug delivery system.^[^ ^6^ ^]^ Exosomes are natural cell‐membrane‐derived nanovesicles that have been extensively investigated due to their structure, composition, and unique properties in intercellular communication. However, the clinical application of exosomes remains limited due to their complex production processes and low yield. With advancements in nanotechnology, cell membrane nanovesicles (CMNs) have been developed using various physical and chemical methods, yielding much higher amounts than naturally secreted exosomes.^[^ ^7^ ^]^ In comparison with exosomes, 100‐fold was achieved in the production yield of CMNs from the same number of cells.^[^ ^8^ ^]^ Therefore, the application of CMNs in drug delivery has greatly improved. Zhang and co‐workers were the first to successfully develop erythrocyte‐membrane‐derived CMNs, which retain many beneficial properties of erythrocyte membrane such as high biocompatibility, ease of operation, and cost‐effectiveness.^[^ ^9^ ^]^ However, limitations such as weak targeting ability and low treatment efficacy remain. To enhance the properties of CMNs, engineering the cell membrane has proven to be an effective method.^[^ ^10^ ^]^

Currently, genetic engineering can be used to selectively express the desired proteins or peptides on cell membranes, enabling further improvement in the targeting ability and therapeutic effects of CMNs.^[^ ^11^ ^]^ Compared to nongenetic modification methods, such as click chemistry, electrostatic interactions, and hydrophobic insertion, genetic engineering has several unique advantages, including the preservation of cell activity and retaining original protein bioactivity on CMNs. By precisely regulating target protein genes, their natural structure, orientation, and complete biological activity can also be maintained. Furthermore, large‐scale production and long‐term storage are possible by creating stable cell lines through genetic engineering.^[^ ^12^ ^]^ Herein, a brief overview of surface engineering strategies for CMNs and the features of various membrane sources are provided. Subsequently, preparation methods of genetically engineered CMNs (GCMNs) are discussed as is the application of GCMNs in cancer immunotherapy targeting different immune targets. Finally, the challenges and prospects of GCMNs in clinical translation are addressed.

## 2 CMN Surface Engineering Strategy

**Figure 1:**
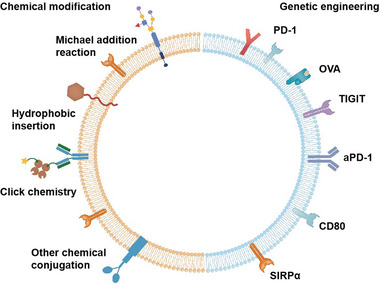
Surface engineering of cell membrane nanovesicles (CMNs) via genetic engineering or chemical modification. PD‐1: programmed cell death protein 1; OVA: ovalbumin; TIGIT: T cell immunoreceptor with immunoglobulin and ITIM domain; aPD‐1: anti‐programmed‐cell‐death‐protein 1 antibody; SIRP*α*: signal regulatory protein alpha.

Due to the lack of targeting ability, CMNs are easy to be eliminated quickly following delivery into the body, resulting in a short half‐life. To maximize the potential of CMNs as delivery carriers, various modification strategies have been developed, the most promising of which is surface engineering.^[^ ^13^ ^]^ Surface engineering encompasses genetic engineering and chemical modifications with the goal of endowing CMNs with additional functions (**Figure** **1**). Furthermore, CMN surface engineering strategies are discussed in **Table** **1**.

**Table 1:** Summary of surface engineering strategies for CMNs. CMNs: cell membrane nanovesicles

Engineering strategies	Bond strength	Advantages	Disadvantages
Genetic engineering	Strong	High transfection efficiency; long‐term storage; large‐scale production; permanent	Operation complex; efficiency instability; high cost and time duration
Covalent chemical modification	Strong	Easy operation; convenient; wide application range	Toxicity; possible damage of structure and biological activity of CMNs
Noncovalent chemical modification	Weak	Easy operation; mild reaction conditions	Toxicity; weak bonding strength; possible structure and biological activity damage of CMNs

### 2.1 Genetic Engineering

Genetic engineering is a widely used technique to modify cells using the cell's own biosynthetic machinery and is recognized as an effective method for the modification of cell membranes.^[^ ^14^ ^]^ By genetically modifying the original cells, PD‐1, PD‐L1, and multiple antibodies have been specifically expressed on the surface of different CMNs.^[^ ^15^ ^]^ GCMNs have been reported to significantly enhance specific targeting ability and therapeutic effects in cells or tissues. For instance, Zhou and co‐workers increased targeting of CMNs by overexpressing chemokine (C‐X‐C motif) receptor 4 (CXCR4) on the surface of neural stem cells, creating a GCMN with enhanced targeting to the ischemic brain and improved delivery efficiency.^[^ ^11b^ ^]^ Similarly, Liu and co‐workers successfully developed biofunctionalized liposome‐like nanovesicles displaying human epidermal growth factor (EGF) or anti‐human epidermal growth factor receptor (anti‐HER2) affibody that actively target cancer cells. Human EGF on the surface of GCMNs had significantly better biological activity and targeting ability than human EGF conjugated onto liposomes through chemical modification.^[^ ^12b^ ^]^ Moreover, genetic modification of CMNs can also exert therapeutic effects. Chen and co‐workers prepared CMNs overexpressing PD‐1 through genetic engineering of TC‐1P cells, and showed that PD‐1 overexpressed on the surface of CMNs were bound to PD‐L1 on the surface of lung cancer cells, exerting anticancer efficacy.^[^ ^16^ ^]^ While genetic engineering is a favorable method to endow CMNs with additional features, various limitations remain – the process of genetic engineering is complex, labor intensive, and unpredictable, and not all cells can be easily genetically modified.

### 2.2 Chemical Modification

Surface molecules of cell membranes, such as amino, carboxyl, and sulfhydryl groups, provide various active sites for chemical modification. Chemical modification can be classified into covalent chemical modifications and noncovalent chemical modifications. CMNs are frequently chemically modified to achieve stable anchoring of proteins on their surface. Generally, chemical modification is convenient, simple, and has a wide application range.^[^ ^17^ ^]^ However, the surface complexity of CMNs may decrease reaction efficiency, and covalent chemical modifications may affect the structure and function of CMNs, with various active chemical functional groups, such as dyes, biotin, and azido groups, likely increasing toxicity. Therefore, future work could focus on finding safe and effective functional groups for chemical modification.

#### 2.2.1 Covalent Chemical Modification

Covalent chemical modification of CMNs involves the introduction of exogenous functional groups and bioactive molecules onto the surface of CMNs by forming chemical bonds. Click chemistry is often used to attach protein peptides and antibodies to the surface of CMNs. For example, Cai and co‐workers enhanced the targeting ability of T‐cell‐membrane‐derived CMNs through click chemistry.^[^ ^18^ ^]^ Michael addition reactions between maleimide and sulfhydryl groups are commonly used to efficiently and selectively modify protein sites without damaging proteins on the surface of CMNs. Cheng and co‐workers introduced functionalized succinimidyl‐[(*N*‐maleimidopropionamido)‐polyethyleneglycol] ester onto the surface of CMNs and coupled thiolated human recombinant hyaluronidase via a thiol–maleimide reaction. Thus, by coupling maleimide with sulfhydryl groups, functional molecules can be introduced onto the surface of CMNs while preserving their structure and function.^[^ ^19^ ^]^

#### 2.2.2 Noncovalent Chemical Modification

The membrane structure of CMNs is, like that of cell membranes, primarily composed of phospholipids, cholesterol, and glycolipids. Amphiphilic substances containing different functional groups can be efficiently embedded into the surface of CMNs driven by hydrophobic interactions. Liu et al. demonstrated that cholesterol‐modified aptamers (short nucleotide sequences that bind to the corresponding ligands with high affinity and strong specificity) could be efficiently embedded into the surface of CMNs through cholesterol, and CMNs engineered by hydrophobic insertion strategies effectively activated anticancer activity.^[^ ^20^ ^]^ In another example, Zhang and co‐workers bound cyclic (Arg–Gly–Asp) peptide onto the surface of CMNs through noncovalent chemical modification, promoting CMN accumulation in tumors.^[^ ^21^ ^]^ As the surface of CMNs is negatively charged, cationic nanocomplexes can be bonded to their surface via electrostatic interactions. Thus, Zhang and co‐workers introduced positively charged nanocomplexes into the cell membrane, achieving targeting capacity and mucus‐penetrating efficiency. However, the low stability of noncovalent binding through electrostatic interaction may impact the efficacy of this method.^[^ ^22^ ^]^

## 3 Cell Membrane Properties Employed in GCMNs

**Figure 2:**
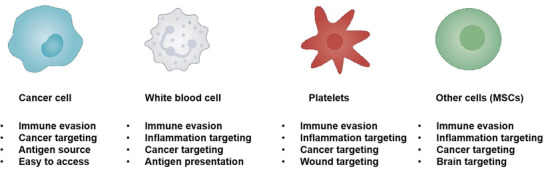
Properties of genetically engineered cell membrane nanovesicles (GCMNs) retained from original cells. MSCs: mesenchymal stem cells.

GCMNs retain the natural properties of cell membranes, such as excellent biocompatibility and negligible immunogenicity, resulting in an extended circulation time in the body. Cell membranes used in GCMNs include outer membranes from leukocytes, platelets, mesenchymal stem cells, cancer cells, and bacterial vesicles.^[^ ^23^ ^]^ The outer membranes of leukocytes, mesenchymal stem cells, and platelets can evade recognition by the immune system due to their inherent properties, whereas outer membranes of bacterial vesicles and cancer cells can stimulate an immune response. Moreover, the various cell membranes have different proteins on their surfaces, forming diverse types of “camouflage coats.” The outer membranes of cancer cells, for instance, have a homologous aggregation function, leading to a homing effect.^[^ ^24^ ^]^ Therefore, GCMNs obtained from genetically engineered cancer cells have good homologous targeting ability, enabling targeted therapy (**Figure** **2**). Accordingly, suitable cell membrane coatings can be selected based on specific application requirements to achieve biological targeting and enhance cancer therapy effects.

Platelets participate in wound healing after tumor surgery and escape phagocytosis by macrophages.^[^ ^25^ ^]^ Zhang et al. engineered megakaryocytes to express PD‐1, producing platelets with stable PD‐1 expression that aggregate at the surgical site. PD‐1 in platelets then binds to PD‐L1 on cancer to reverse cytotoxic T lymphocyte (CD8^+^ T cell) activity, reducing postoperative tumor recurrence and metastasis.^[^ ^26^ ^]^ DCs are “professional” antigen‐presenting cells (APCs) capable of processing and presenting antigens to initiate T‐cell‐mediated immune responses.^[^ ^27^ ^]^ Furthermore, major histocompatibility complex (MHC) class I (MHC‐I) molecules on the surface of DCs play a critical role in inducing specific cytotoxic T cell activation.^[^ ^28^ ^]^ With the above in mind, Liu et al. used viral vectors to transduce antigen genes into DCs, inducing antigen expression on the surface of DCs. During this process, DCs are stimulated by the virus, resulting in high levels of MHC‐I on the cell membrane surface. GCMNs thus prepared can be used as biomimetic APCs to activate a strong antitumor immune response.^[^ ^23a^ ^]^

The unique characteristics of cancer cells can be attributed to the complex antigenic features of their membranes, allowing for specific recognition of homologous cancer cells.^[^ ^29^ ^]^ The cancer cell membrane performs the “localization” function by adhering to and recognizing other cancer cells, enabling cancer‐cell‐derived GCMNs to accumulate on the surface of cancer cells. T cells and macrophages possess various membrane receptors and specific surface proteins that exhibit exploitable properties such as long blood circulation, easy crossing of biological barriers, and recognition of cancer lesions.^[^ ^30^ ^]^ Li and co‐workers constructed T cells with high PD‐1 expression via genetic engineering and obtained GCMNs designed to utilize the original PD‐1 properties of T cells to better ensure the expression efficiency of PD‐1 for therapeutic effects.^[^ ^31^ ^]^ In another example, Wang and co‐workers genetically engineered macrophages to express PD‐1 for immunotherapy of brain gliomas.^[^ ^32^ ^]^ Thus, given the effect of cell‐membrane‐intrinsic features on GCMN properties, it is important to select the appropriate membrane materials for the desired outcomes.

## 4 Preparation of GCMNs

**Figure 3:**
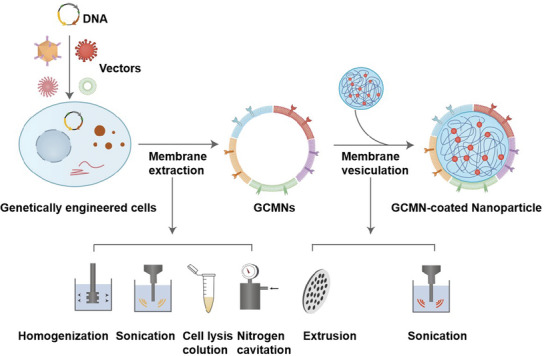
General preparation process of GCMNs.

GCMN preparation involves genetic engineering of the candidate cell and cell membrane extraction and vesiculation (**Figure** **3**).

### 4.1 Methods for the Genetic Engineering of Cell Membranes

In recent decades, advances in genetic engineering have greatly improved cancer treatment.^[^ ^33^ ^]^ The central step in this process involves the delivery of gene vectors carrying genes into candidate cells. Existing gene vector delivery systems mainly employ viral (retroviral, adenoviral, and lentiviral vectors) and nonviral vectors (liposomes, polymers, and nanoparticles)^[^ ^34^, ^35^ ^]^ (**Table** **2**).

**Table 2:** Viral vectors and nonviral vectors used in genetic engineering

Type of vectors	Options (examples)	Advantages	Disadvantages
Viral vectors	Retroviral vectors, adenoviral vectors, and lentiviral vectors	High transfection efficiency; maintain the integrity of protein‐intrinsic structure and orientation; permanent	Complex preparation; limited loading capacity; high cost
Nonviral vectors	Liposomes, polymers, and nanoparticles	Adequate loading capacity; low risks; low cost; stable reagent; and easy to store	Low transfection efficiency; toxicity; possible damage of structure and biological activity of cells

#### 4.1.1 Viral Vectors

Viral vectors are among the most widely studied vectors due to their wide host range and high transfection efficiency. However, their wide use is currently hindered due to their limited packaging capacity and complex preparation.^[^ ^36^ ^]^ After decades of development, viral vectors – retroviral, adenoviral, and lentiviral vectors – have shown promising results in clinical trials.^[^ ^37^ ^]^

Retroviral vectors were designed based on the characteristics of retroviruses. Additionally, retroviral vectors efficiently enter target cells, and are therefore widely used in genetic engineering.^[^ ^14^ ^]^ The primary advantage of retroviral vectors is their ability to effectively integrate exogenous genes into human target cell genomes, leading to stable and persistent gene expression.^[^ ^38^ ^]^ However, they are limited by their ability to infect only dividing cells and the potential to activate oncogenes or insert mutations, leading to carcinogenic risk.^[^ ^39^ ^]^

Adenoviral vectors are excellent gene delivery vehicles and efficient high‐capacity vectors in genetic engineering.^[^ ^40^ ^]^ They differ from retroviral vectors in that they can infect both dividing and nondividing cells.^[^ ^41^ ^]^ Adenoviral vectors have high titers, a wide host range, excellent safety, and the ability to be transformed into oncolytic viruses for cancer therapy. However, they do not integrate into host chromosomes, can only provide transgene expression for a short duration, and they do not replicate with host cell division, requiring repeated transfection.^[^ ^42^ ^]^

Lentiviral vectors are the most commonly used vector type in genetic engineering^[^ ^43^ ^]^ and belong to the retrovirus family;^[^ ^44^ ^]^ however, in contrast to retroviral vectors, lentiviral vectors are more complex.^[^ ^45^ ^]^ Lentiviral vectors contain glycoproteins on their surface, allowing them to transfect a variety of cell types as well as both dividing and nondividing target cells, making them suitable for various gene delivery applications.^[^ ^46^ ^]^ Lentiviral vectors can effectively integrate exogenous genes into host chromosomes, enabling persistent expression of these genes.

Another important viral vector is adeno‐associated viral vectors.^[^ ^47^ ^]^ The gene delivery efficiency of adeno‐associated viral vectors is largely dependent on the molecular interaction between the surface protein capsid and the target cell‐surface receptor.^[^ ^48^ ^]^

#### 4.1.2 Nonviral Vectors

Nonviral vectors use the physical and chemical properties of synthetic vector materials to mediate gene transfer.^[^ ^49^ ^]^ The mechanism of gene delivery by nonviral vectors mainly involves electrostatic interactions between positively charged vectors and negatively charged nucleic acid molecules, resulting in the formation of nanoparticles (NPs).^[^ ^50^ ^]^ NP formation can protect nucleic acid fragments from enzymatic degradation during transport, retaining the positive charge effects that promote electrostatic interactions between NPs and the cell membrane surface, facilitating the transport of nucleic acids into cells. Compared with viral vectors, nonviral vectors have a larger packaging capacity, controllable structure, lower cost, and simple preparation methods.^[^ ^51^ ^]^ However, their application remains limited due to their low transfection, specificity, and safety, although improvements of these properties in recent decades have led to increased usage of nonviral vectors in clinical trials.^[^ ^35^, ^52^ ^]^ Currently investigated nonviral vectors include liposomes, polymers, and NPs.^[^ ^53^ ^]^

Cationic liposomes (with a cationic headgroup, linker, and hydrophobic moieties) typically consist of cationic lipids and neutral helper lipids. The cationic headgroup plays a critical role in the electrostatic interaction between nucleic acid molecules and cationic liposomes, and linker length can impact the interaction between cationic liposomes and the cell membrane, thereby affecting transfection efficiency.^[^ ^54^ ^]^ Cationic liposomes are widely used as gene delivery carriers due to their protection of nucleic acids from degradation and provision of specificity to target cells, their biodegradability, and their ease of preparation and capacity to deliver large DNA fragments into cells.^[^ ^55^ ^]^

Cationic polymer carriers are polymer compounds with positive charge structure unit.^[^ ^56^ ^]^ Cationic polymers are classified into natural and synthetic polymers based on their source – natural polymers include proteins, peptides, and polysaccharides, while synthetic polymers include polyethylene glycol amine, chitosan, and poly(amidoamine).^[^ ^57^ ^]^ Cationic polymers, such as chitosan, interact with DNA via electrostatic interactions, preventing DNA molecule degradation and ensuring their integrity within cells. As a gene carrier, chitosan has low cytotoxicity, good biocompatibility, low immunogenicity, and high gene transfection efficiency.^[^ ^58^ ^]^ The major difference between cationic polymers and cationic liposomes is that the former are entirely water‐soluble, lack hydrophobic portions, and can be easily chemically modified.^[^ ^59^ ^]^

NPs, including silicon, iron oxide, carbon nanotubes, calcium phosphate, metal NPs, and quantum dots, can also be used as gene delivery carriers.^[^ ^60^ ^]^ The most significant advantage of inorganic materials is their controllable size and shape, making it possible to study the impact of these features on transfection efficiency. While their transfection efficiency as gene vectors may be lower than that of viral vectors, they have considerable advantages, including high specific surface area, large loading capacity, high stability, easy storage, convenient preparation, and low toxicity.^[^ ^61^ ^]^ For example, gold NPs have been extensively utilized in gene delivery due to their unique chemical and optical properties, ease of surface modification, and enhanced permeability.^[^ ^62^ ^]^ Additionally, NPs can directly treat disease by delivering drugs or biomolecules across cell membranes and into cells.^[^ ^63^ ^]^

Overall, genes transfected by nonviral vectors do not integrate into the chromosomes of target cells and do not pose a risk of insertion mutation, making them potentially safer than viral vectors. However, nonviral vectors have disadvantages such as insufficient targeting, toxicity, low transfection efficiency, and short effective expression time.^[^ ^64^ ^]^

### 4.2 Cell Membrane Extraction and Vesiculation

Following the modification of cells via genetic engineering, GCMNs can be prepared in a similar manner to traditional CMNs, namely through cell membrane extraction and cell membrane vesiculation.^[^ ^23^, ^65^ ^]^ The first step in cell membrane extraction is cell lysis, which can be achieved through mechanical homogenization, sonication, cell lysis solution, or nitrogen cavitation. The resulting homogenate mixture is then separated by differential or gradient centrifugation to obtain cell membranes. High‐purity cell membranes are obtained by purification through sucrose gradient centrifugation. Finally, GCMN vesiculation is achieved through coextrusion and sonication. Thus, the prepared GCMNs maintain basic biological properties similar to the original cell membranes albeit in nanoscale dimensions. During the vesiculation process, various NPs can also be loaded to achieve additional functions; for example, poly(lactic‐*co*‐glycolic acid) (PLGA) NPs, magnetic iron oxide NPs, and polyethylene glycol NPs have previously been loaded onto GCMNs.^[^ ^18^, ^66^ ^]^ The interfacial interaction between the surface charge of NPs and cell membranes is crucial in determining effective loading of NPs onto GCMNs.^[^ ^67^ ^]^ Negatively charged NPs and membrane shells allow for successful membrane coating, constructing a right‐side‐out orientation that ensures proper presentation of proteins in the external environment. Conversely, when the cell membrane is mixed with positively charged NPs, it becomes challenging to form a stable shell–core structure due to strong electrostatic attractions leading to rapid aggregation.^[^ ^68^ ^]^

#### 4.2.1 Coextrusion

Coextrusion is a common method for the preparation of GCMNs. In coextrusion, a series of porous polycarbonate films with progressively smaller pore sizes are utilized to prepare uniform GCMNs.^[^ ^69^ ^]^ For example, a GCMN was extracted via cell lysis solution and sonication, followed by the preparation of nanovaccines loaded with PLGA NPs.^[^ ^70^ ^]^ The coextrusion method has the advantages of producing uniform GCMN‐coated NPs, ensuring a bioactive membrane, and a wide application range. However, limitations include complicated operation, time‐consuming procedures, and the possible loss of cell membrane integrity.

#### 4.2.2 Sonication

GCMNs can also be produced by sonication. Cavitation bubbles generated by ultrasound waves can damage the cell membrane structure and promote NP reassembly with the surrounding membranes.^[^ ^8^ ^]^ Both chemically modified CMNs and GCMNs can be obtained by ultrasound.^[^ ^71^ ^]^ For instance, Zhang and co‐workers mixed an extracted cell membrane with PLGA and used sonication to prepare a delivery system.^[^ ^65^ ^]^ Compared to coextrusion, sonication has advantages such as involving a one‐step reaction, less material loss, and obtaining a high loading efficiency (**Table** **3**). However, due to the high energy generated by the sonication process, the structure and function of cell membranes may be damaged. Therefore, coextrusion may be more suitable for unstable samples.

**Table 3:** Methods for preparing genetically engineered‐cell‐membrane‐nanovesicle‐coated nanosystems

Preparation method	Principle of the technique	Advantages	Disadvantages
Coextrusion	Mechanical force deforms the cell membrane and promotes nanoparticles to cross the phospholipid bilayer of membrane vesicles	Ensures bioactivity of membrane; uniform particle size; wide application range	Complex operation; time‐consuming; possible loss of cell membrane integrity
Sonication	Ultrasonic waves facilitate the reassembly of membranes around nanoparticles	One‐step reaction; low loss of material; high loading efficiency	Nonuniform particle size; irreversible damage; possible loss of cell membrane integrity

Following the preparation of GCMNs, the completeness and integrity of GCMNs can be evaluated via various methods, including transmission electron microscopy, mass spectrometry protein analysis, dynamic light scattering analysis, stability assays, and binding exclusion assays.

## 5 Application of GCMNs in Cancer Immunotherapy

**Figure 4:**
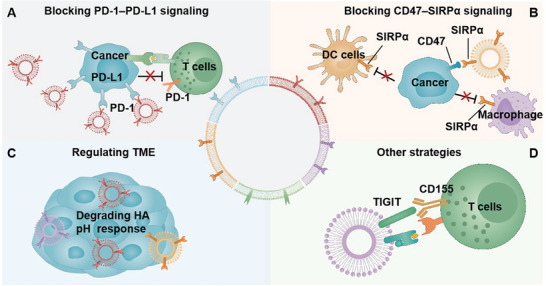
Application of GCMNs in immunotherapy. GCMNs have shown promising application in cancer immunotherapy by acting on immune targets and stimulating immune responses. GCMNs can achieve effective immune responses through A) blocking of programmed cell death protein 1 (PD‐1)–PD‐L1 signaling: GCMNs overexpressing PD‐1 competitively bind to PD‐L1 and effectively block PD‐1–PD‐L1 signaling; B) blocking CD47–signal regulatory protein alpha (SIRP*α*) signaling: GCMNs overexpressing SIRP*α* competitively bind to CD47 and effectively block CD47–SIRP*α* signaling; C) regulating the tumor microenvironment (TME): GCMNs can regulate the TME by acting on special targets such as by expressing hyaluronidase to degrade hyaluronic acid (HA), thus destroying TEM composition; D) other strategies such as GCMNs overexpressing T cell immunoreceptor with immunoglobulin and ITIM domain (TIGIT) and blocking TIGIT–CD155 signaling. DC: dendritic cell.

GCMNs have significant effects in cancer immunotherapy, and these have been widely reported. The combination of genetic engineering technology and biomimetic nanotechnology provides GCMNs with unique functions and characteristics. GCMNs target various cancer‐related signaling pathways and functional proteins on their surfaces can be exploited for treatment and diagnosis. Additionally, GCMNs can be combined with other therapeutic methods through their excellent encapsulation properties to enhance the efficacy of antitumor therapy. Herein, we focus on the application of GCMNs in cancer immunotherapy (**Figure** **4**). **Table** **4** lists selected examples of GCMNs for cancer immunotherapy.

**Table 4:** Examples of genetically engineered cell membrane nanovesicles employed in cancer immunotherapy. PD‐1: programmed cell death protein 1; MSCs: mesenchymal stem cells; aPD‐L1: anti‐programmed‐cell‐death‐ligand 1 antibody; OVA: ovalbumin; aPD‐1: anti‐programmed‐cell‐death‐protein 1 antibody; SIRP*α*: signal regulatory protein alpha; MMP‐2: matrix metallopeptidase‐2; HA: hyaluronic acid; ECM: extracellular matrix; VSVG: vesicular stomatitis virus G‐protein; TRAIL: tumor‐necrosis‐factor‐associated apoptosis‐inducing ligand; TIGIT: T cell immunoreceptor with immunoglobulin and ITIM domain; gCM‐MNs: genetically engineered cell‐membrane‐coated magnetic nanoparticles; BHK‐21: baby hamster kidney‐21; HEK293T: human embryonic kidney 293T cells; TNFα: tumor necrosis factor–α; IFNγ: interferon–γ

Applications	Name	Membrane source	Genetic vectors	Engineering protein	Therapeutic target	Refs.
Blocking PD‐1–PD‐L1 signaling	1‐MT@PD1‐NVs	HEK293T cells	Mammalian expression vector	PD‐1	T cells	[74]
	PD‐1‐MM@PLGA/RAPA	Macrophages	Lentivirus vector	PD‐1	T cells	[32]
	AAI–R837	MSCs	Lentivirus vector	aPD‐L1 and OVA	T cells, DC cells	[23b]
	CPI‐444–aPD‐1‐scFv‐NVs	Macrophages	Lentivirus vector	aPD‐1	T cells	[77]
	ASPIRE	DC cells	Adenovirus vector and plasmid	OVA and aPD‐1	T cells	[23a]
Blocking CD47–SIRP*α* signaling	gCM‐MNs	Tumor cells	Lentivirus vector	SIRP*α*	Macrophage	[84]
	Fus‐CVs	Tumor cells	Lentivirus vector and mammalian expression vector	SIRP*α* and PD‐1	Macrophage, T cells, DC cells	[85]
	DBE@CCNPs	Tumor cells	GoldenTran‐S	CD47 knockout	Antigen‐presenting cells	[87]
Regulating TME	mHAase@nP18	BHK‐21 cells	Lentivirus vector	MMP‐2–HAase	ECM	[92]
	Lp‐KR‐CCM‐A	Tumor cells	Plasmid	KillerRed	Tumor cells	[95]
	SPN‐TF	HEK293T cells	Plasmid	Transferrin	Tumor cells	[97]
	Tf@IR820–DHA	HEK293T cells	–	Transferrin	Tumor cells	[99]
	MVVs—N3	HEK293T cells	Lentivirus vector	VSVG	TME	[101]
Other types	TM–CQ/NPs	LX2 cells	Lentivirus vector	TRAIL	Tumor cells	[105]
	[CD80/OVA]NPs	Tumor cells	Plasmid and retroviral expression vector	CD80 and OVA	T cells	[65]
	O‐TPNVs	HEK293T cells	Lentivirus vector	TIGIT	Tumor cells	[110]
	IL‐15/IL‐15R*α*‐NVs	NIH 3T3 cells	Lentivirus vector	IL‐15/IL‐15R*α*	T cells	[113]

### 5.1 Blocking PD‐1–PD‐L1 Signaling of GCMNs

PD‐1 is an immune‐checkpoint receptor mainly expressed by activated T cells.^[^ ^72^ ^]^ Cancer cells express PD‐L1 and bind to PD‐1 on T cells. Upon PD‐1–PD‐L1 binding, negative regulatory signals are transmitted to T cells, leading to failure in recognizing cancer cells and allowing immune escape.^[^ ^73^ ^]^ Blocking PD‐1–PD‐L1 binding can activate T cells and result in the elimination of cancer cells. Several studies have focused on the preparation of GCMNs able to block the PD‐1–PD‐L1 signaling pathway for cancer therapy.

#### 5.1.1 GCMNs Expressing PD‐1

**Figure 5:**
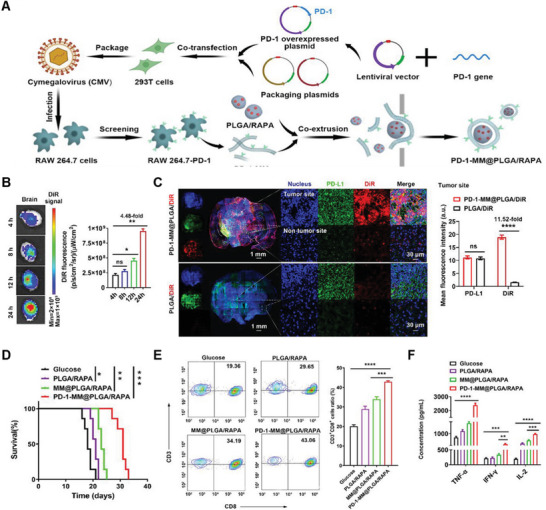
Anticancer genetically engineered cell membrane nanovesicles fabricated by overexpressing PD‐1. A) PD‐1 was introduced into RAW 264.7 cells using lentiviral vector and the cell membrane was coextruded with PLGA/RAPA to obtain PD‐1‐MM@PLGA/RAPA. B) DiR signals in isolated mice brains at different time points after intravenous (i.v.) injection of PD‐1‐MM@PLGA/DiR. C) Representative fluorescence images of PLGA/DiR and PD‐1‐MM@PLGA/DiR at 24 h after i.v. injection. D) Survival rates of mice treated with glucose, PLGA/RAPA, MM@PLGA/RAPA, and PD‐1‐MM@PLGA/RAPA. E) Population of CD8^+^ cytotoxic T lymphocytes within the cancer. F) Quantification of TNF*α*, IFN*γ*, and IL‐2. Reproduced with permission.^[^ ^32^ ^]^ Copyright 2022, American Chemical Society.

GCMNs expressing PD‐1 can actively target PD‐L1 on the surface of cancer cells and prevent PD‐1–PD‐L1 signaling between T cells and cancer cells, revitalizing T cells and eradicating cancer cells. Zhang et al. genetically engineered HEK293T cells to stably express the mouse PD‐1 receptor and obtained PD‐1‐receptor‐expressing CMNs, blocking the PD‐1–PD‐L1 immunosuppression axis to enhance cancer immunotherapy and effectively restore depleted CD8^+^ T cells able to attack cancer cells. Moreover, PD‐1‐receptor‐expressing CMNs can be combined with a small‐molecule inhibitor of indoleamine 2,3‐dioxygenase to further limit cancer cell proliferation.^[^ ^74^ ^]^ HEK293 cells are often used as original cells for GCMN preparation due to their relatively easy transfection characteristics. However, in various studies in mouse models, HEK293‐cell‐derived vesicles increase immunogenicity. Therefore, many experiments use murine‐derived cells. Glioblastoma has a unique tumor immune microenvironment and is challenging to treat due to the blood–brain barrier. Considering this, Wang and co‐workers used a lentiviral vector encoding the *PD1* gene for transfection of macrophages, which overexpressed PD‐1 (RAW 264.7–PD‐1). Macrophage cell membranes overexpressing PD‐1 (PD‐1‐MM) were coated onto PLGA NPs loaded with rapamycin (PLGA/RAPAs) to obtain PD‐1‐MM@PLGA/RAPA (**Figure** **5**A). The in vivo targeting and bioactivity of PD‐1‐MM@PLGA were tested after intravenous injection, recording their biological distribution and accumulation in the brain. The accumulation of PD‐1‐MM@PLGA/DiR in the brain moderately increased over time, reaching a maximum at 24 h postinjection (Figure 5B). Compared to PLGA/DiR, the average fluorescence intensity of PD‐1‐MM@PLGA/DiR increased by 11.52 times, indicating that PD‐1‐MM@PLGA/RAPA NPs could cross the blood–brain barrier and specifically aggregate at tumor sites with high PD‐L1 expression (Figure 5C). The treatment effect was then studied in mice, and the group treated with PD‐1‐MM@PLGA/RAPA showed a significantly longer survival than other groups (Figure 5D). Thus, PD‐1‐MM@PLGA/RAPA significantly increased the proportion of tumor‐infiltrating CD8^+^ cytotoxic T lymphocytes, increased the expression levels of cytokines TNF*α*, IFN*γ*, and interleukin (IL)‐2, and improved the immunosuppressive microenvironment compared to other groups (Figure 5E,F).^[^ ^32^ ^]^ Furthermore, PD‐1 was expressed on the surface of macrophage cell membranes, and the derived GCMNs played a significant curative effect. However, considering the unique advantages of exosomes in the blood–brain barrier in glioblastoma, future work could use exosomes as delivery carriers for diagnosis and treatment. Besides macrophages, T cells and TC‐1 cell lines can also be genetically engineered to construct stable PD‐1‐expressing cells and the corresponding GCMNs expressing PD‐1 for cancer immunotherapy.^[^ ^16^, ^31^ ^]^

#### 5.1.2 GCMNs Expressing Anti‐PD‐1

**Figure 6:**
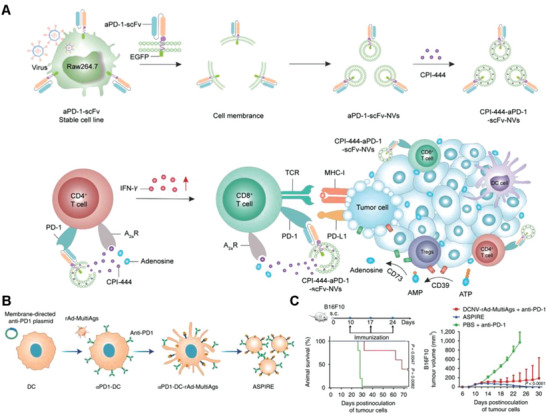
Anticancer genetically engineered cell membrane nanovesicles fabricated by overexpressing anti‐PD‐1. A) Preparation of CPI‐444–aPD‐1‐scFv‐NVs and anticancer mechanism of CPI‐444–aPD‐1‐scFv‐NVs. Reproduced with permission.^[^ ^77^ ^]^ Copyright 2022, Tsinghua University Press. B) Preparation of ASPIRE. C) Survival rates and tumor growth curves in mice under different treatments. Reproduced with permission.^[^ ^23a^ ^]^ Copyright 2022, Springer Nature Limited.

In addition to expressing PD‐1 protein to competitively block the PD‐1–PD‐L1 immunosuppressive axis, monoclonal antibody drugs can also be used to block this pathway.^[^ ^75^ ^]^ Although monoclonal antibodies have achieved significant clinical efficacy, PD‐1 monoclonal antibody therapy has a low clinical response rate and can easily lead to an autoimmune response and other toxic side effects.^[^ ^76^ ^]^ Therefore, novel anti‐PD‐1 antibody delivery systems are currently being developed. Zhang and co‐workers genetically engineered mouse macrophages to express PD‐1 single‐chain antibodies, extracted cell membranes to prepare CMNs (aPD‐1‐scFv‐NVs: nanovesicles displaying anti‐programmed cell death‐1 single‐chain variable fragment antibody), and loaded the A2a adenosine receptor antagonist (CPI‐444) to prepare CPI‐444–aPD‐1‐scFv‐NVs (**Figure** **6**A).^[^ ^77^ ^]^ GCMNs not only display target proteins on the surface but can also achieve cotransfer of different target proteins for multifunctional efficacy. For example, Liu et al. successfully constructed GCMNs that can directly activate both naive and exhausted T cells, promoting functional remodeling of exhausted T cells and effectively reversing cancer immunosuppression. The cell membrane of DCs was genetically engineered to express anti‐PD‐1, and cell membrane isolation resulted in the formation of GCMNs that integrates antigen self‐presentation and immunosuppression reversal (ASPIRE) (Figure 6B). ASPIRE demonstrated the best antitumor effect than other groups in the B16F10 subcutaneous tumor model (Figure 6C).^[^ ^23a^ ^]^ Although the production of ASPIRE is complicated, it has a wide range of clinical applications, such as in the treatment of chronic viral infection, proving great advantages in clinical translation.

#### 5.1.3 GCMNs Expressing Anti‐PD‐L1

**Figure 7:**
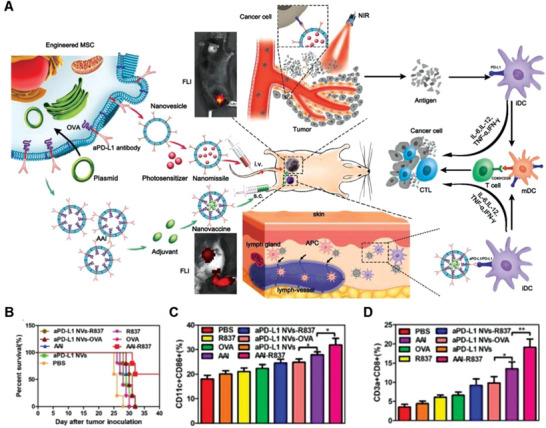
Anticancer genetically engineered cell membrane nanovesicles fabricated by overexpressing anti‐PD‐L1 (aPD‐L1). A) Preparation of aPD‐L1 NVs–indocyanine green (ICG) and antigen–antibody integrator (AAI). Following intravenous injection of aPD‐L1 NVs–ICG, the photosensitized agent was delivered to the tumor by targeting PD‐L1. The primary tumor was laser irradiated to induce reprogramming. Subsequently, subcutaneous injection of AAI in mice enhanced the antitumor immune response. B) Survival rates with different treatments. C) Rates of mature DCs in mouse lymph nodes after the last dose for 3 days. D) Percentage of CD8^+^ T cells in the distal secondary tumor after the last dose for 3 days. Reproduced with permission.^[^ ^23b^ ^]^ Copyright 2022, Springer Nature.

The delivery PD‐L1 monoclonal antibody drugs to block the PD‐1–PD‐L1 signaling axis is a promising strategy in anticancer treatment.^[^ ^78^ ^]^ Liu and co‐workers designed two GCMNs that express anti‐PD‐L1 for this purpose. The first method used a lentiviral vector to infect bone marrow mesenchymal stem cells, construct cell lines expressing anti‐PD‐L1 antibodies (aPD‐L1), and extract and isolate membrane NVs expressing aPD‐L1 (aPD‐L1 NVs). aPD‐L1 NVs were further loaded with indocyanine green, significantly improving the efficacy of photothermal ablation and synergistically enhancing subsequent immune effects targeting tumors (**Figure** **7**A). The second method involved cotransfecting antigenic ovalbumin (*OVA*) and *aPD‐L1* genes into bone marrow mesenchymal stem cells, and extracting the cell membrane to prepare the derived antigen antibody integrator (AAI). AAI was then loaded with Imiquimod (R837) to construct the nanovaccine AAI–R837 (Figure 7A). aPD‐L1 binds to DCs and delivers the immune adjuvant, where the antigenic signal is recognized by the corresponding DCs, inducing a powerful immune response. Following immunization with the different substances, the growth and survival of distal tumors were closely monitored. Data showed that AAI–R837 had the best tumor inhibition of all groups and cured 60% of tumors in mice (Figure 7B). In addition, mature DC and cytotoxic T lymphocyte numbers in the mice treated with AAI–R837 were higher than in mice in other treatment groups, indicating the best curative effect (Figure 7C,D).^[^ ^23b^ ^]^ However, in addition to tumor cells, PD‐L1 receptors are also widely expressed in normal cells or tissues, such as liver cells, vascular endothelium, mesenchymal stem cells, and muscle, leading to serious immune‐related adverse events. Thus, Liang et al. recently constructed M‐*α*PD‐L1 NVs expressing a matrix metallopeptidase‐2 (MMP‐2)‐activating lock masking on *α*PD‐L1 (M‐*α*PD‐L1) through genetic engineering, which can avoid *α*PD‐L1 binding with normal tissues.^[^ ^114^ ^]^ Furthermore, high MMP‐2 expression in tumors cleaves the curling peptides of the MMP‐2‐activating lock, exposing *α*PD‐L1 and blocking the PD‐1–PD‐L1 signaling axis at the cancer site, providing a promising strategy to enhance cancer immunotherapy. Similarly, Wang and co‐workers obtained CMNs overexpressing PD‐L1 through genetic engineering and coated them on PLGA NPs to prepare mesenchymal stem cell (MSC)–PD‐L1^+^ NPs used to reduce immune‐related adverse events induced by ICIs.^[^ ^79^ ^]^ CAR‐T cell therapy has achieved significant clinical efficacy in leukemia and other hematological cancers. However, the clinical efficacy in solid cancers remains unsatisfactory. In order to enhance the efficacy of CAR‐T cells in solid cancers, Cao and co‐workers developed GCMNs expressing high‐affinity aPD‐L1 loaded with glutamine antagonists for cancer immunotherapy.^[^ ^80^ ^]^

### 5.2 Blocking CD47–Signal Regulatory Protein Alpha (SIRP*α*) Signaling of GCMNs

CD47 belongs to the immunoglobulin superfamily and is expressed on the surface of various cancer cells, with SIRP*α* on macrophages serving as its ligand.^[^ ^81^ ^]^ Cancer cells are thought to avoid being phagocytosed through expression CD47, thereby activating a “do not eat me” signaling pathway that enables immune escape for cancer cells.^[^ ^82^ ^]^ Consequently, blocking the CD47–SIRP*α* inhibitory signal can promote macrophage phagocytosis of cancer cells.

#### 5.2.1 GCMNs Expressing SIRP*α*

Effectively activating the innate immune system to kill cancer, particularly through macrophage‐mediated cancer cell phagocytosis, is a promising strategy. CD47 on the surface of cancer cells binds to SIRP*α* on macrophages to inhibit macrophage‐mediated phagocytosis. CD47‐blocking antibodies (anti‐CD47) can prevent this inhibition and promote macrophage‐mediated phagocytosis of cancer cells.^[^ ^83^ ^]^ However, monoclonal antibodies are easily cleared by the body, thus leading to higher therapeutic dose requirements to overcome drug degradation caused by anti‐CD47. Additionally, given the widespread expression of CD47, anti‐CD47 may also lead to off‐target effects, and thus to side effects such as anemia. To address these issues, Chen and co‐workers developed a hybrid CMN system comprising platelet‐derived NVs, M1‐macrophage‐derived NVs, and genetically engineered cancer‐cell‐derived NVs that overexpress high‐affinity SIRP*α* variants. These hybrid CMNs retain the functions of the source cells, effectively accumulating at the surgical site, and interacting with circulating tumor cells in the blood. Furthermore, hybrid CMNs repolarize tumor‐associated macrophages into the M1 phenotype, blocking the CD47–SIRP*α* signaling pathway, enhancing macrophage‐mediated phagocytosis of cancer cells, improving T cell immunity against cancer, and reducing systemic‐infusion‐induced side effects.^[^ ^84^ ^]^

**Figure 8:**
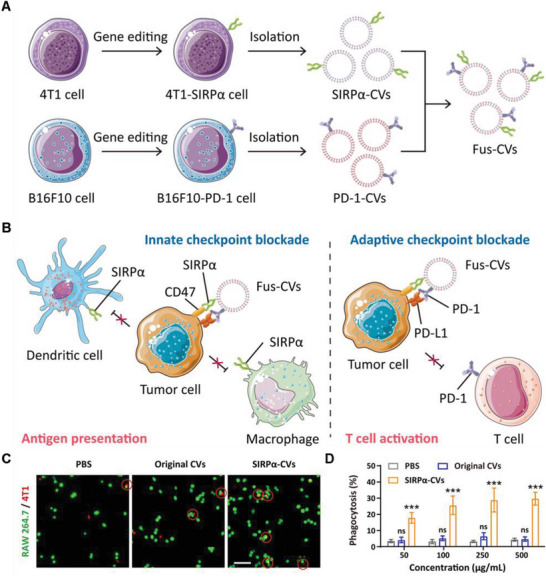
Anticancer genetically engineered cell membrane nanovesicles fabricated by overexpressing SIRP*α*. A) Preparation of Fus‐CVs. B) Anticancer mechanism of Fus‐CVs. C) Fluorescence images of phagocytosis assays. Scale bar, 100 µm. D) Quantitative analysis of 4T1 cell phagocytosis by RAW264.7 cells at different culture concentrations. Reproduced with permission.^[^ ^85^ ^]^ Copyright 2021, Wiley‐VCH.

Targeting multiple cancer immune checkpoints simultaneously is more effective in restricting cancer cell escape from immune surveillance compared to targeting a single checkpoint. Considering this, Rao and co‐workers developed a genetically engineered fused cellular vesicle (Fus‐CV) system for dual‐targeting therapy. First, cellular vesicles (CVs) containing SIRP*α* (SIRP*α*‐CVs) and PD‐1 (PD‐1‐CVs) were severally obtained through genetic engineering. Fus‐CVs simultaneously displaying SIRP*α* and PD‐1 were then obtained using fusion technology (**Figure** **8**A). The double targeting capability of Fus‐CVs can block the innate immune checkpoint CD47 and the adaptive immune checkpoint PD‐L1, promoting antigen presentation by macrophages and DCs, and triggering an antitumor T cell immune response (Figure 8B). To investigate whether SIRP*α*‐CVs can induce phagocytosis by macrophages, 4T1 cells were treated with either original CVs or SIRP*α*‐CVs, then cocultured with RAW 264.7 macrophages. Confocal images showed that the phagocytic ability of macrophages was significantly improved after treatment with SIRP*α*‐CVs (Figure 8C,D). Fus‐CVs that display high‐affinity SIRP*α* variants and PD‐1 for dual‐targeting ICI therapy showed better targeting ability and therapeutic effects than original CVs.^[^ ^85^ ^]^ Furthermore, in Fus‐CVs, the CVs can be designed for the required purpose. Despite current work targeting CD47 and PD‐L1 checkpoints, the Fus‐CV platform could be extended to simultaneously target other checkpoints to synergize cancer immunotherapy.

#### 5.2.2 CD47‐Knockout GCMNs

**Figure 9:**
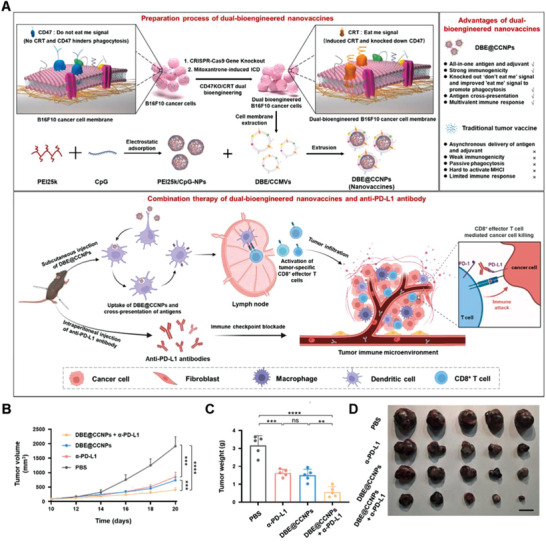
Anticancer genetically engineered cell membrane nanovesicles fabricated by CD47 knockout. A) Preparation of DBE@CCNPs and anticancer mechanism of DBE@CCNPs. Tumor B) volume and C) weight profiles after the various treatments in different groups. D) Tumor images after the various treatments in different groups. Scale bar, 1 cm. Reproduced with permission.^[^ ^87^ ^]^ Copyright 2022, Elsevier.

CMNs derived from cancer cells have shown promise in cancer vaccine immunotherapy. However, cancer cells often evade the immune system through CD47 upregulation, which binds to SIRP*α* on DCs and transmits a negative signal.^[^ ^86^ ^]^ To enhance the ability of cancer vaccines to induce DC maturation and antigen cross‐presentation, Liu et al. developed a nanovaccine constructed using CD47 knockout (CD47KO)/calreticulin (CRT) dual‐bioengineered B16F10 tumor cell membranes and an unmethylated cytosine–phosphate–guanine (CpG) adjuvant. Clustered regularly interspaced short palindromic repeat/CRISPR‐associated nuclease 9 (CRISPR–Cas9) gene editing technology was used to knock out CD47 in vitro and construct CD47 knockout B16F10 cells (CD47KO‐B16F10). Mitoxantrone was then used to induce immunogenic cell death in CD47KO‐B16F10 cells, resulting in CRT translocation on the surface of CD47KO‐B16F10 cells. Cell membranes of CD47KO/CRT dual‐bioengineered tumor cells were extracted and coextruded with polyetherimide with relative molecular mass of 25k (PEI25k)/CpG NPs to prepare the CD47KO/CRT dual‐bioengineered cancer‐cell‐membrane‐coated NPs (DBE@CCNPs), which promoted the endocytosis of antigens and adjuvants in mouse‐bone‐marrow‐derived DCs, effectively stimulating APCs and activating specific T cells, thereby eliciting an antitumor immune response (**Figure** **9**A). DBE@CCNPs significantly inhibited tumor growth (Figure 9B–D), indicating triggering of effective antitumor immunity.^[^ ^87^ ^]^ Additionally, some studies have shown that CD47 knockout tumor cells can be effectively phagocytized by primary macrophages, leading to tumor size reductions.^[^ ^88^ ^]^

### 5.3 GCMNs Regulating the Tumor Microenvironment (TME)

The TME consists of cellular and noncellular components surrounding tumor cells, and its composition changes dynamically with tumor occurrence and development.^[^ ^89^ ^]^ The complexity and diversity of the TME present many obstacles to the immunotherapy of solid cancers.^[^ ^90^ ^]^ In recent years, many studies have focused on improving therapeutic efficacy by targeting and reshaping the TME, which can be regulated in various ways, including through alterations in its structure and targeting of TME‐associated signaling molecules.^[^ ^91^ ^]^

#### 5.3.1 GCMNs Expressing MMP‐2‐Responsive hyaluronidase (mHAase)

**Figure 10:**
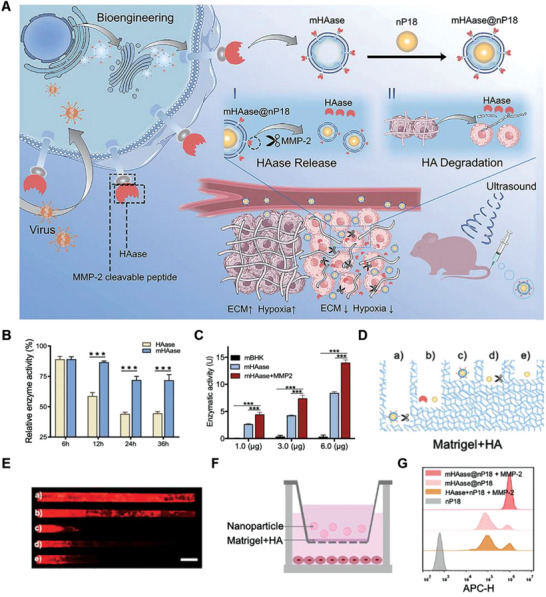
Anticancer genetically engineered cell membrane nanovesicles fabricated by expressing mHAase. A) The preparation and antitumor mechanism of mHAase@nP18. B) Enzyme activity profiles of mHAase and HAase in serum at 37 °C for 6, 12, 24, and 36 h (*n* = 3). C) HAase activity of HAase on vesicles and release responding to MMP‐2 (*n* = 3). D) Schematic diagram of ECM‐like capillary models. E) Confocal laser‐scanning microscopy of nP18 diffusion in ECM‐simulation gels, a) mHAase@nP18 + MMP‐2; b) HAase + nP18; c) mHAase@nP18; d) nP18 + MMP‐2; e) nP18. Red (P18). F) The Transwell‐based TME model. G) Flow cytometry plots showing nP18 penetration of HepG2 cells after the various treatments. Reproduced with permission.^[^ ^92^ ^]^ Copyright 2022, Wiley‐VCH.

The dense extracellular matrix (ECM) is an important acellular component of TME that impedes the efficacy of anticancer therapy. Multiple studies have shown that ECM remodeling plays an important role in regulating tumor immunity. Tumor‐associated ECM can be targeted therapeutically in various ways, including altering the structure or physical properties of the ECM. Hyaluronic acid (HA) is a major component of the ECM. Therefore, strategies targeting HA to modulate the TME have been developed, mostly by utilizing hyaluronidases (HAases), enzymes that degrade HA. For example, Liu and co‐workers designed a NV system with a high expression of mHAase. mHAase was further loaded with the sonosensitizer, purpurin 18 (P18), to fabricate mHAase@nP18 for dual‐mode fluorescence/photoacoustic‐image‐guided sonodynamic therapy (**Figure** **10**A). The activity of mHAase hyaluronidase was evaluated, and date showed that commercial‐free HAase was rapidly inactivated, while HAase expressed on vesicles significantly maintained its activity in serum for 36 h (Figure 10B). More importantly, HAase released in response to MMP‐2 cleavage had higher enzymatic activity than mHAase, possibly due to its properties as a secreted protein (Figure 10C). The authors also designed an ECM‐like capillary model to evaluate the drug diffusion of mHAase@nP18 by HA degradation, and showed that mHAase@nP18 had the most significant degradation effect on HA (Figure 10D). Similarly, the red fluorescence of mHAase@nP18 + MMP‐2 group filled with the whole capillary, which was stronger than that of the HAase + nP18 group, mHAase@nP18 without MMP‐2 cleavage, and the other control groups, indicating that the mHAase@nP18 + MMP‐2 had better penetration and diffusion ability (Figure 10E). To simulate the delivery order of mHAase@nP18 in the TME, a Transwell‐based TME model was constructed by conducting an ECM simulation in superstratum and uptake process of HepG2 cells in substratum (Figure 10F). After incubation for 6 h, the cells in the lower layer of the Transwell plate were collected and analyzed by flow cytometry. Compared with other groups, mHAase + MMP‐2 had the highest uptake in HepG2 cells, further indicating that mHAase + MMP‐2 damaged the ECM and played an important role in enhancing drug uptake in tumor cells (Figure 10G).^[^ ^92^ ^]^ HAase cannot be expressed through gene modification in nonnucleated cells such as red blood cells; therefore, to modify HAase on the surface of red blood cells, chemical modification can be adopted. For example, Cheng and co‐workers chemically modified human recombinant hyaluronidase PH20 on red blood cell membranes and extracted the membrane coated with NPs to prepare a drug delivery system. In the extracellular HA matrix model, the drug delivery system also effectively disrupted the matrix to assist the diffusion of NPs.^[^ ^19^ ^]^

#### 5.3.2 GCMNs Expressing KillerRed (KR)

**Figure 11:**
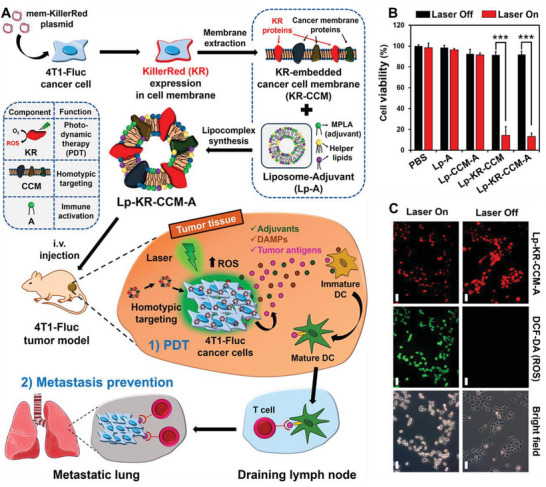
Anticancer genetically engineered cell membrane nanovesicles fabricated by expressing KR. A) The preparation and anticancer mechanism of Lp‐KR‐CCM‐A. B) Viability of cancer cells treated with phosphate‐buffered saline (PBS) or different groups with or without laser irradiation for 1 h. C) In vitro reactive oxygen species (ROS) generation induced by Lp‐KR‐CCM‐A upon laser irradiation for 20 min. Scale bars, 50 µm. Reproduced with permission.^[^ ^95^ ^]^ Copyright 2019, American Chemical Society.

Photodynamic therapy (PDT) is a promising therapeutic approach to trigger cancer cell death by using photosensitizers to produce reactive oxygen species (ROS) through photochemical reactions.^[^ ^93^ ^]^ However, most chemical photosensitizers have poor biocompatibility and physical properties that limit their effectiveness in cancer therapy.^[^ ^94^ ^]^ KR protein is a novel photosensitizer that can produce ROS under green light irradiation, showing strong therapeutic potential in cancer treatment. To improve the efficiency of KR targeting of cancer cells, KR was genetically engineered to be expressed on the cancer cell membrane (KR‐CCM). Monophosphoryl lipid A, a lipid adjuvant, was embedded in a liposome (Lp‐A) and KR‐CCM and Lp‐A membranes were fused to create Lp‐KR‐CCM‐A. This approach achieved high cancer targeting efficiency due to the homomorphic affinity of CCM to the source cancer cells. KR‐embedded Lp‐KR‐CCM‐A produced cytotoxic ROS during PDT, effectively inducing an anticancer immune response and inhibiting primary cancer growth and lung metastasis in homotype cancer‐bearing mice (**Figure** **11**A). Furthermore, various lipid complexes were tested, with Lp‐KR‐CCM and Lp‐KR‐CCM‐A showing highly photoactivated cytotoxicity compared to those not containing KR, indicating the vital role of KR proteins in PDT (Figure 11B). Following laser irradiation, Lp‐KR‐CCM‐A produced cytotoxic ROS and resulted in morphological changes such as cell detachment, demonstrating its therapeutic potential for cancer treatment (Figure 11C).^[^ ^95^ ^]^ Overall, this study shows the promise of using genetically engineered KR protein to enhance the efficacy of PDT for cancer treatment, potentially overcoming the limitations of chemical photosensitizers.

#### 5.3.3 GCMNs Expressing transferrin (Tf)

**Figure 12:**
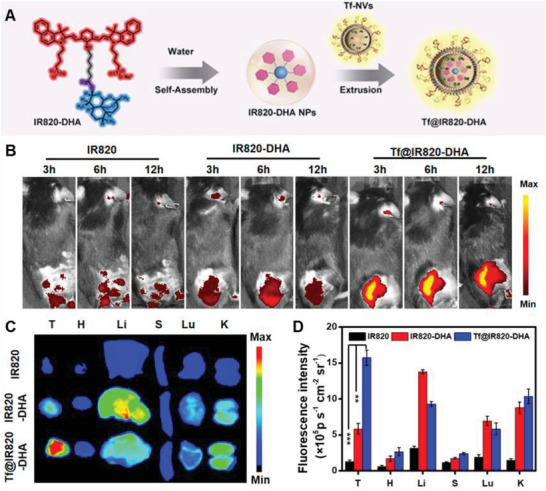
Anticancer genetically engineered cell membrane nanovesicles fabricated to express transferrin (Tf). A) The preparation of Tf@IR820–DHA. B) In vivo near‐infrared fluorescence imaging of tumors in mice upon injection of different treatments via the tail vein after 3, 6, and 12 h. C) Ex vivo fluorescence imaging of tumors and major organs after 6 h of treatment. D) The corresponding fluorescence intensity of tumors and major organs after 6 h of treatment (*n* = 3). Reproduced with permission.^[^ ^99^ ^]^ Copyright 2022, American Chemical Society.

The transferrin receptor (TfR) allows iron to enter cells and is often overexpressed on the surface of cancer cells, particularly in metastatic and drug‐resistant cancers.^[^ ^96^ ^]^ TfR has been widely used to improve the targeted effect of drug delivery systems to cancer cells by identifying overexpressed receptors on the cell surface. Therefore, GCMNs targeting the TfR of cancer cells have been developed as a powerful approach for targeted therapy. Guo and co‐workers successfully extracted a genetically engineered cell membrane with high Tf expression (CM‐TF) and transformed a novel D–A‐conjugated polymer into water‐soluble nanoparticles using amphiphilic polymer 1,2‐distearoyl‐sn‐glycero‐3‐phosphoethanolamine‐N‐[methoxy(polyethylene glycol)‐2000] (DSPE‐PEG2000). CM‐TF was then coated onto the surface of SPN to create the photothermal conversion agent SPN‐TF, capable of targeting cancer.^[^ ^97^ ^]^ Furthermore, Tf has also been used to deliver iron effectively.^[^ ^98^ ^]^ For instance, Liu and co‐workers prepared GCMNs expressing Tf and encapsulated IR820–dihydroartemisinin NPs (IR820–DHA) to construct Tf@IR820–DHA (**Figure** **12**A), which targets tumor tissue in vivo. Compared with free IR820 and IR820–DHA NPs, Tf@IR820–DHA showed higher fluorescence intensity in tumor areas and demonstrated effective targeting ability (Figure 12B). Moreover, 6 h after injection, Tf@IR820–DHA exhibited a greater concentration of tumor sites than free IR820 and IR820–DHA NPs, with even higher fluorescence levels in tumor tissues compared to other major organs (Figure 12C) and ≈15 and 3 times higher, respectively, than tumors treated with IR820 and IR820–DHA NPs (Figure 12D). Thus, Tf@IR820–DHA has an improved tumor targeting ability, effectively delivering iron and IR820–DHA NPs to the tumor site. Overall, GCMNs expressing Tf offer a promising approach for targeted therapy in cancer treatment.^[^ ^99^ ^]^ Although Tf@IR820–DHA has successfully been used to target primary and distant tumor models in mice, further studies in primary carcinoma and large animal models are needed.

#### 5.3.4 GCMNs Expressing Vesicular Stomatitis Virus G‐Protein (VSVG)

**Figure 13:**
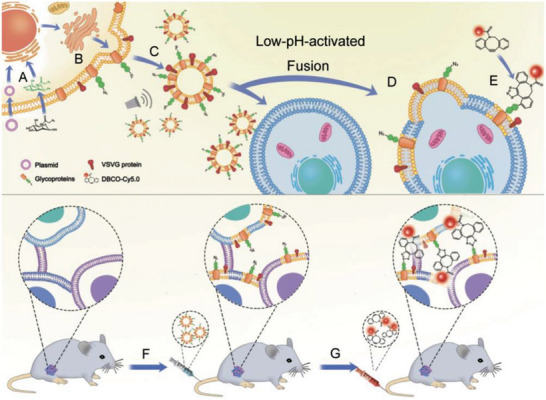
A–G) Preparation of mimovirus vesicle with azide motifs (MVVs—N3) for cancer diagnosis. Reproduced with permission.^[^ ^101^ ^]^ Copyright 2020, Wiley‐VCH.

The heterogeneity of tumors creates significant challenges for both tumor diagnosis and treatment. While active targeting strategies based on tumor‐cell‐specific ligand molecules, such as antibodies and peptides, are effective for tumor diagnosis, variability in the expression of natural receptors in tumor cells can limit the implementation of this approach.^[^ ^100^ ^]^ The spike VSVG effectively promotes plasma membrane fusion of two or more adjacent cells under acidic conditions, which is the hallmark of solid tumors. Liu and co‐workers employed genetic engineering to express VSVG in HEK293T cells, and then used the cell membranes to produce mimovirus vesicles (MVVs). These MVVs were modified by attaching small‐molecule receptor azide motifs (—N3) to their surface. As a pH‐responsive functional protein, the VSVG protein responds to the slightly acidic environment found in tumors, promoting cell membrane fusion, while —N3 enables efficient binding and enrichment in vivo and in vitro with dibenzocyclooctyne‐modified small molecules or nanodrug carriers through a biological click reaction (**Figure** **13**). Thus, MVVs respond to the slightly acidic TME and promote plasma membrane fusion.^[^ ^101^ ^]^ Further studies are needed with other cell models given the immunogenicity problems of HEK293T cells.

### 5.4 Other Types of GCMNs

#### 5.4.1 GCMNs Expressing CD80

**Figure 14:**
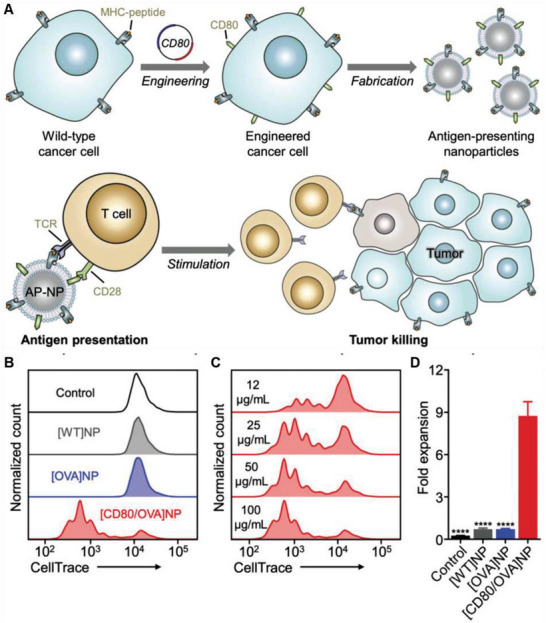
Anticancer genetically engineered cell membrane nanovesicles fabricated by expressing OVA and CD80. A) The preparation and anticancer mechanism of [CD80/OVA] (NPs). B,C) Fluorescent signal dilution of CD8^+^ T cells in a population of OT‐I splenocytes after incubation with different groups (B) or [CD80/OVA] NPs at various concentrations (C) for 3 days. D) Fold expansion of CD8^+^ T cells in a population of OT‐I splenocytes after incubation with different groups for 4 days (*n* = 3). Reproduced with permission.^[^ ^65^ ^]^ Copyright 2020, Wiley‐VCH.

T cell activation requires two stimulatory signals – the T cell receptor (TCR) signal formed by the combination of TCR and the antigenic peptide–MHC complex, which is in itself not enough to activate T cells, and the costimulatory signal provided by the combination of CD28 and CD80, required to fully activate T cells.^[^ ^102^, ^103^ ^]^ To achieve better anticancer efficacy by fully activating T cells, Zhang and co‐workers engineered cancer cells to express CD80 costimulatory signals, generating a B16–CD80 cell line. Retroviral vectors were then used to transfer *OVA* genes into B16–CD80 cells, producing B16–CD80/OVA cells. The cell membranes of B16–CD80/OVA cells were extracted and PLGA NPs were coated to prepare antigen‐presenting [CD80/OVA] NPs (**Figure** **14**A). The study showed that [CD80/OVA] NPs induced a significant amount of cell division, whereas the other groups had a minimal effect on cell proliferation (Figure 14B). Moreover, the proliferative effect was dependent on the [CD80/OVA] NP concentration, and [CD80/OVA] NPs at 100 µg mL^−1^ led a majority of T cell proliferation (Figure 14C). Indeed, cells treated with [CD80/OVA] NPs proliferated ninefold within 4 days, while the number of cells in other control samples decreased. The T cell activation properties of the [CD80/OVA] NPs were also confirmed (Figure 14D).^[^ ^65^ ^]^ In this study, CD80 and OVA were engineered to construct a biomimetic nanoscale APC platform, which directly presented tumor antigens to activate T cells for tumor therapy. It is also notable that the present study was generated without other immunostimulatory compounds such as adjuvants, ICIs, or cytokines; therefore, future studies should include these to enhance treatment potency.

#### 5.4.2 GCMNs Expressing Tumor‐Necrosis‐Factor‐Associated Apoptosis‐Inducing Ligand (TRAIL)

**Figure 15:**
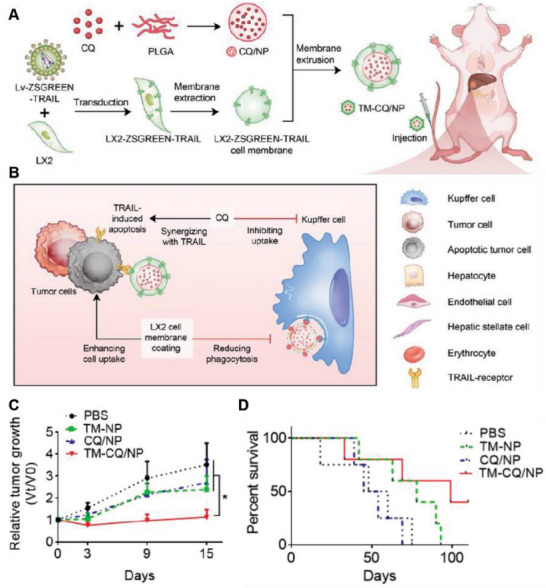
Anticancer genetically engineered cell membrane nanovesicles fabricated by expressing TRAIL. A,B) Preparation and antitumor mechanism of TM–CQ/NPs. C) Tumor growth curves in mice with different treatments. D) Survival rates in nude mice with different treatments (*n* = 4 or 5). Reproduced with permission.^[^ ^105^ ^]^ Copyright 2022, Elsevier.

TRAIL is a promising anticancer active molecule because of its ability to selectively bind to proapoptotic death receptors, which are frequently overexpressed in a wide range of tumor cells, subsequently inducing apoptosis in these cells.^[^ ^104^ ^]^ However, the use of TRAIL is limited due to instability, easy removal, and requiring repeated dosing to maintain the effective concentration of TRAIL in cancer. To overcome these problems, various methods have been developed to improve the bioavailability of TRAIL and overcome resistance. Liu et al. constructed liver fibroblast cell lines (LX2) that reliably express TRAIL and obtained the cell membrane expressing TRAIL protein (TM). Chloroquine (CQ), a clinical autophagy inhibitor, was coated onto PLGA NPs to prepare CQ/NP, which were finally coated with TM to prepare (TM–CQ/NP). TM–CQ/NP specifically induced apoptosis of tumor cells through the binding of TRAIL protein and its death receptor but had little effect on normal cells. CQ further inhibited the uptake of TM–CQ/NP by macrophages and collaboratively induced tumor cell apoptosis with TRAIL protein (**Figure** **15**A,B). In mouse tumor models of in situ hepatocellular carcinoma and colorectal cancer peritoneal metastasis, TM–CQ/NP accumulated in tumor tissue and had excellent antitumor activity (Figure 15C,D).^[^ ^105^ ^]^ In addition to using cell membranes as vectors for genetically engineered TRAIL expression, bacterial membranes can also be used as a delivery vector. Ning and co‐workers obtained derived outer membrane vesicles by expressing TRAIL on the surface of *Escherichia coli* using genetic engineering techniques. Studies have shown that TRAIL on the bacterial outer membrane vesicles surface also plays an important role in inducing apoptosis of cancer cells.^[^ ^106^ ^]^

#### 5.4.3 GCMNs Expressing T Cell Immunoreceptor with Immunoglobulin and immunoreceptor tyrosine‐based inhibitory motif (ITIM) Domain (TIGIT)

**Figure 16:**
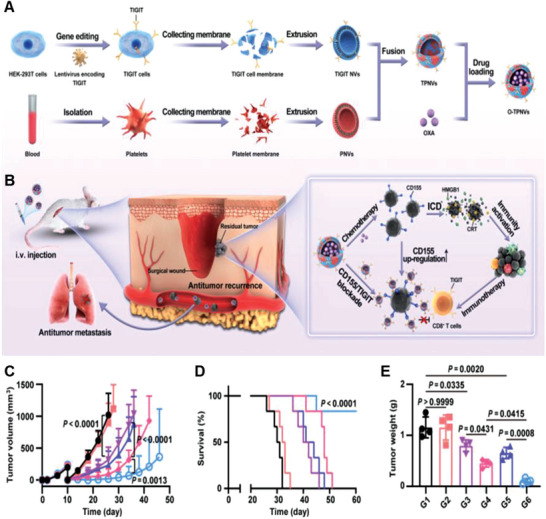
Anticancer genetically engineered cell membrane nanovesicles fabricated to express TIGIT. A,B) Preparation and anticancer mechanism of O‐TPNVs. C) Recurrent cancer growth curves after different treatments (*n* = 6). D) Survival rates in mice after different treatments (*n* = 6). E) Weight of recurrent cancers after different treatments (*n* = 4). Reproduced with permission.^[^ ^110^ ^]^ Copyright 2022, American Association for the Advancement of Science.

CD155 is a crucial cell adhesion molecule that plays a significant role in cancer progression by regulating signaling pathways related to cell proliferation, migration, invasion, and adhesion.^[^ ^107^, ^108^ ^]^ CD155 interacts with the TIGIT receptor on immune cells, leading to inhibition of the T‐cell‐ or natural‐killer‐cell‐mediated immune response. Researchers have developed several drugs targeting CD155–TIGIT signaling albeit with single‐drug approaches with efficacy limitations.^[^ ^109^ ^]^ Therefore, studies exploring combined drug administration approaches, such as chemotherapy and immunotherapy, to achieve better treatment outcomes are ongoing. Mei and co‐workers used fusion NVs with TIGIT‐expressing cell membrane and platelet cell membrane (TPNVs) loaded with oxaliplatin (OXA) to create a drug delivery system (O‐TPNVs). The platelet‐derived membrane components in O‐TPNVs effectively targeted postoperative cancer wounds. OXA directly killed residual cancer cells, induced immunogenic cell death, and activated the immune system. TIGIT of O‐TPNVs bound to CD155 on the surface of cancer cells, blocked CD155–TIGIT signaling, and restored the activity of CD8^+^ T cells (**Figure** **16**A,B). Thus, this combination therapy effectively inhibited postoperative cancer recurrence and metastasis and prolonged overall survival (Figure 16C–E). Active targeting of the platelet membrane on the surface of O‐TPNVs, along with OXA release, promoted immunogenic cell death of cancer cells and activated the anticancer immune response. Additionally, TIGIT on the surface of O‐TPNVs blocked the CD155–TIGIT pathway, eliminating possible immune escape. This approach represents an innovative and ingenious combination therapy.^[^ ^110^ ^]^ The results of a clinical study of anti‐TIGIT combined with anti‐PD‐1 in the treatment of non‐small‐cell lung cancer are encouraging, indicating the potential of TIGIT for cancer therapy. Therefore, PD‐1 and TIGIT could be simultaneously displayed on the surface of GCMNs, which needs to be further studied.

#### 5.4.4 GCMNs Expressing IL‐15/IL‐15R*α*

**Figure 17:**
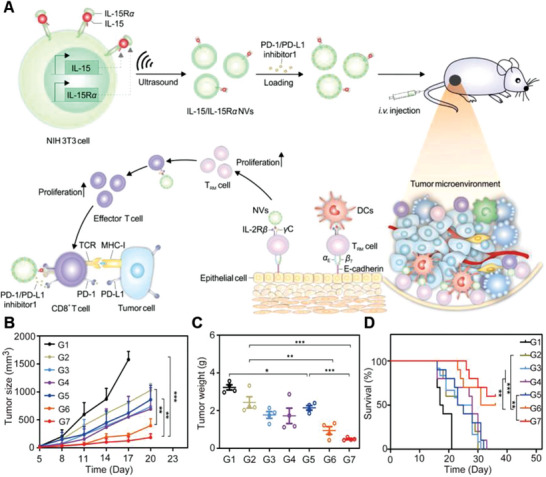
Anticancer genetically engineered cell membrane nanovesicles fabricated to express IL‐15/IL‐15R*α*. A) The preparation and anticancer mechanism of IL‐15/IL‐15R*α* NVs–PD‐1/PD‐L1 inhibitor 1. B) Recurrent cancer growth curves after different treatments (*n* = 5). C) Weight of recurrent tumors after different treatments (*n* = 10). D) Survival rates in mice after different treatments (*n* = 4). Reproduced with permission.^[^ ^113^ ^]^ Copyright 2022, Elsevier.

Interleukin‐2 was the first biological immune agent of the interleukin family cytokines used in tumor therapy.^[^ ^111^ ^]^ In recent years, other interleukins have also shown promise in cancer therapy, including IL‐15, which plays an important role as a biological response regulator of the immune system.^[^ ^112^ ^]^ However, the clinical application of IL‐15 is limited by its short half‐life and instability in the blood and its lack of cancer targeting ability. To address these challenges, Zhang and co‐workers used genetic engineering to stably express the IL‐15/IL‐15R*α* complex in NIH 3T3 cells and obtained IL‐15/IL‐15R*α* NVs. PD‐1/PD‐L1 inhibitor 1 was then loaded into the IL‐15/IL‐15R*α* NVs to create the drug delivery system IL‐15/IL‐15R*α* NVs–PD‐1/PD‐L1 inhibitor 1 (**Figure** **17**A). In a tumor model, IL‐15/IL‐15R*α* NVs–PD‐1/PD‐L1 inhibitor 1 treatment effectively delayed tumor growth and further enhanced the antitumor response, inhibited tumor growth, and improved the survival rate of mice (Figure 17B–D).^[^ ^113^ ^]^

## 6 Conclusions and Perspectives

GCMNs obtained from genetically engineered original cells through physicochemical methods can exert varying anticancer effects. This review summarizes, for the first time, progress in GCMN development for anticancer immunotherapy from different immune targets as well as the effects produced by GCMNs, including blocking PD‐1–PD‐L1 signaling, blocking CD47–SIRP*α* signaling, and regulating the TME. GCMNs have gained attention due to their unique advantages, and numerous studies in recent years have yielded impressive results. Due to the superiority of genetic engineering and convenience for large‐scale CMN production, GCMNs will become a promising strategy for cancer immunotherapy in the future.

Despite progress in the field of GCMNs for cancer immunotherapy, several challenges need to be addressed before their translation into the clinic, including the selection of candidate cells for GCMNs and the availability, abundance, and in vitro culture conditions of candidate cells. For example, the features of cell membranes at different growth phases and cell cycle stages may result in batch‐to‐batch variation, which could affect the effectiveness of treatment. Furthermore, it is critical to ensure immunocompatibility since GCMNs will likely be produced from allogeneic membrane source materials. Two potential avenues to address this concern are the selection of autologous cells or patient‐derived induced pluripotent stem cells as the cell membrane source, or engineering universal cell lines in which potentially immunogenic antigens are genetically knocked out. Autologous cells are ideal materials to reduce the risk of host immune responses. However, the timely availability for preparation of GCMNs, involving a multistep isolation process and rigorous quality control procedures, is a limitation. By contrast, engineered universal cell lines can provide a ready cell source. Therefore, cell sources and related standard operating procedures should be the focus of future research. Furthermore, the availability of safe and effective gene delivery vectors remains a barrier to genetic engineering of cells. Recent breakthroughs have been demonstrated through the modification of cells by delivering specific messenger ribose nucleic acids (mRNAs) directly to candidate cells to obtain GCMNs.

From a manufacturing perspective, another vital link is the large‐scale preparation of GCMNs. The yield, purity, and homogeneity of GCMNs after preparation would be critically influenced by processing variables. However, thus far, research on this topic is minimal and should be the focus of future studies. Furthermore, GCMNs, as biological agents, require storage in a frozen state, which may compromise the integrity of the membrane. Therefore, studies should also focus on related cryoprotectants to minimize membrane damage during storage.

Overall, to meet the needs of clinical applications, GCMN preparation must be improved, including standard operating procedures, safety and efficacy evaluation, and storage and transportation. Despite these limitations, the field of GCMNs is rapidly growing, and researchers are working to address these challenges. For example, new techniques for the production of CMNs are being developed, and efforts are under way to improve their safety. As more research is conducted on GCMNs, these challenges will eventually be overcome and their clinical application in cancer therapy is likely to be achieved.

## Conflict of Interest

The authors declare no conflict of interest.
